# Epigenetic modifications and immune responses in HIV-infected infants: a narrative review

**DOI:** 10.1097/MS9.0000000000003142

**Published:** 2025-03-07

**Authors:** Emmanuel Ifeanyi Obeagu

**Affiliations:** Department of Biomedical and Laboratory Science, Africa University, Mutare, Zimbabwe

**Keywords:** biomarkers, epigenetic modifications, immune dysregulation, pediatric HIV/AIDS, therapeutic strategies

## Abstract

HIV infection in infants poses unique challenges due to the interplay between an immature immune system and the viral mechanisms that exploit it. Recent research has unveiled critical insights into how epigenetic modifications, such as DNA methylation, histone modifications, and noncoding RNA regulation, specifically influence immune responses in HIV-infected infants. These modifications are not merely passive markers of infection but active players in immune dysregulation, contributing to persistent immune activation and the skewing of T-cell differentiation. Emerging studies have highlighted that these epigenetic alterations may play a role in the heightened vulnerability of HIV-infected infants to opportunistic infections and their variable responses to antiretroviral therapy (ART). A growing body of evidence suggests that epigenetic changes in key immune regulatory genes are significantly different in HIV-infected infants compared to uninfected controls. These differences have been linked to altered expression of cytokines, impaired T-cell functionality, and chronic inflammation, which are pivotal in disease progression. Specifically, recent findings indicate that persistent DNA methylation changes in genes involved in T-cell exhaustion could be a major driver of the reduced efficacy of ART in some infants, potentially leading to long-term immune system impairment. Moreover, novel insights into how miRNAs modulate the immune environment in these infants suggest potential targets for therapeutic intervention, aiming to enhance immune recovery and reduce viral reservoirs.

## Introduction

Epigenetic changes refer to heritable modifications in gene expression that do not involve alterations in the underlying DNA sequence. These modifications serve as a regulatory layer, dictating how genetic information is utilized in various cellular contexts. Among these, histone modifications play a pivotal role in epigenetic regulation. Histones, the protein components of chromatin, undergo various posttranslational modifications, such as acetylation, methylation, phosphorylation, and ubiquitination. These modifications influence chromatin structure, transitioning it between an open, transcriptionally active state and a closed, repressive state, thereby controlling gene expression^[1,2]^. In the context of HIV infection, epigenetic modifications contribute to both viral latency and immune dysregulation. Latent HIV reservoirs persist in resting cluster of differentiation 4 (CD4)+ T cells, partly due to epigenetic silencing of the viral genome through histone deacetylation and DNA methylation. These reservoirs pose a major obstacle to achieving a functional cure, especially in pediatric populations where the immune system is still developing. Beyond latency, epigenetic changes also impact host immune responses, including T-cell activation, differentiation, and exhaustion, which are critical in HIV pathogenesis^[3,4]^. Studies have shown that HIV infection disrupts normal epigenetic programming in infants, particularly within immune cells. For example, altered histone methylation patterns in CD4+ and CD8+ T cells have been linked to immune exhaustion, a hallmark of chronic HIV infection. In addition, DNA methylation changes in cytokine and chemokine genes suppress the immune system’s ability to mount effective antiviral responses. These findings highlight the profound impact of HIV on the epigenome of infected infants, which can have lasting effects on their immune development^[5,6]^.

**HIGHLIGHTS**

- Epigenetic modifications impact immune responses in HIV-infected infants.
- DNA methylation and histone modifications regulate key immune genes.
- Noncoding RNAs influence HIV replication and immune function.
- Epigenetic therapies show promising therapeutic potential.
- Understanding epigenetics could improve HIV treatment strategies in infants.

Emerging research suggests that noncoding RNAs, including microRNAs (miRNAs) and long noncoding RNAs (lncRNAs), also play a significant role in modulating immune responses in HIV-infected infants. Dysregulated miRNAs, such as miR-29a and miR-150, have been implicated in impaired immune signaling pathways, while lncRNAs influence T-cell differentiation and function. These epigenetic regulators further complicate the immune landscape in HIV-infected infants, making it crucial to understand their specific roles^[7,8]^. One of the most intriguing findings in recent literature is the epigenetic basis of innate immune memory, also known as trained immunity, in HIV-infected infants. Trained immunity involves epigenetic reprogramming of innate immune cells, such as monocytes and macrophages, leading to enhanced responses upon reexposure to pathogens. However, in HIV-infected infants, this process is often disrupted, resulting in inadequate or hyperactive immune responses. These disruptions are linked to altered histone acetylation and methylation patterns in key immune genes^[9,10]^. Histone modifications also play a role in modulating inflammation in HIV-infected infants. Hyperacetylation of inflammatory gene promoters can lead to excessive inflammation, contributing to immune activation and tissue damage. Conversely, hypoacetylation in antiviral genes suppresses effective immune defense mechanisms. These opposing effects highlight the dual role of histone modifications in balancing immune responses during HIV infection^[5,11]^. Epigenetic interventions, such as histone deacetylase inhibitors (HDACis) and DNA methyltransferase inhibitors (DNMTis), have shown promise in addressing these abnormalities. These therapies aim to reactivate latent HIV while restoring normal immune function. However, their application in infants remains limited due to concerns about off-target effects and long-term safety. Current research is focused on refining these approaches to enhance their specificity and efficacy in pediatric populations[12].

## Aim

This review aims to synthesize current knowledge and research findings on epigenetic modifications in HIV-infected infants, highlighting their role in immune dysfunction, viral persistence, and therapeutic responses.

## Research gap

While significant advancements have been made in understanding the role of epigenetic modifications in the immune responses of HIV-infected infants, several critical gaps remain. Most existing studies focus on cross-sectional data, providing a snapshot of epigenetic changes at a single time point. There is a lack of longitudinal studies that track the evolution of these epigenetic modifications over time in HIV-infected infants. Current research has identified broad patterns of DNA methylation, histone modifications, and noncoding RNA dysregulation in HIV-infected infants. However, there is insufficient knowledge about the specificity of these changes to different immune cell types and how these specific modifications affect cell function. Detailed cell-type-specific epigenetic profiling is necessary to unravel the precise mechanisms at play. While associations between epigenetic modifications and immune dysfunction in HIV-infected infants have been established, the underlying mechanisms remain poorly understood. There is a need for mechanistic studies that explore how specific epigenetic changes lead to altered gene expression and how these alterations contribute to the overall immune response in the context of HIV. The interaction between genetic predispositions and epigenetic modifications in HIV-infected infants has not been fully explored. While potential epigenetic therapies have been proposed, there is a gap in translating these findings into clinical applications. The safety, efficacy, and long-term effects of epigenetic drugs in infants are largely unexplored. Additionally, there is a need to develop biomarkers that can reliably monitor the impact of these therapies on epigenetic modifications and immune function.

## Rationale

The rationale for conducting this review on epigenetic modifications and immune responses in HIV-infected infants stems from the urgent need to address the unique challenges posed by HIV infection in this vulnerable population. Infants who acquire HIV perinatally or through breastfeeding face distinct difficulties compared to adults, largely due to their developing immune systems and the potential for long-term health complications. Infants’ immune systems are immature, making them particularly susceptible to infections and less capable of mounting effective immune responses. HIV infection further complicates this scenario by targeting and impairing key components of the immune system, leading to rapid disease progression and high mortality rates if left untreated. Given these factors, there is a pressing need to understand how epigenetic modifications – a critical layer of gene regulation – affect immune responses in HIV-infected infants. Epigenetic modifications, such as DNA methylation, histone modifications, and noncoding RNAs, play essential roles in regulating gene expression without altering the underlying DNA sequence. These modifications can significantly impact viral replication, latency, and the host’s immune response. In the context of HIV infection, epigenetic changes can influence the expression of immune-related genes, potentially affecting the ability to control the virus and the overall progression of the disease.

Research in adults has demonstrated that epigenetic modifications are involved in the regulation of HIV latency and reactivation, as well as in modulating immune responses. However, there is a relative paucity of studies focusing specifically on infants. Given the critical period of immune development in early life, understanding how these epigenetic changes affect HIV-infected infants could provide valuable insights into disease mechanisms and identify new therapeutic targets. The exploration of epigenetic modifications opens up promising avenues for therapeutic interventions. Epigenetic therapies, including DNA methylation inhibitors, HDACis, and miRNA modulators, have shown potential in reactivating silenced genes, enhancing immune responses, and reducing viral reservoirs. These therapies could be particularly beneficial for HIV-infected infants, who require tailored treatment strategies due to their unique physiological and immunological needs. This review aims to consolidate existing knowledge on the interplay between epigenetic modifications and immune responses in HIV-infected infants, highlighting current research gaps and potential therapeutic strategies. By focusing on this specific population, the review seeks to provide a comprehensive understanding of how epigenetic mechanisms contribute to disease progression and how they can be targeted to improve health outcomes.

## Review methodology

### Literature search

A thorough literature search was conducted using several electronic databases, including PubMed, Google Scholar, and Web of Science, to identify peer-reviewed articles, reviews, and relevant studies. Keywords and search terms used included “HIV-infected infants,” “epigenetic modifications,” “DNA methylation,” “histone modifications,” “noncoding RNAs,” “immune responses,” and “epigenetic therapies.” Boolean operators were employed to refine the search results and ensure a comprehensive capture of relevant literature.

### Study selection

The initial search yielded a substantial number of articles. Titles and abstracts were screened to assess their relevance to the research question. Studies were included if they focused on HIV-infected infants and discussed epigenetic modifications in the context of immune responses. Exclusion criteria involved studies that did not address the specific age group (infants), were unrelated to HIV or epigenetics, or were not available in English.

### Quality assessment

To ensure the reliability and validity of the review, a quality assessment of the included studies was conducted. This involved evaluating the methodological rigor, sample size, and relevance of the studies. High-quality studies were prioritized in the synthesis to provide robust and credible conclusions.

### Epigenetic mechanisms in immune responses

Epigenetic mechanisms play a pivotal role in regulating immune responses during HIV infection in infants, influencing the differentiation, activation, and functional capabilities of immune cells[13]. These mechanisms involve modifications to DNA, histones, and noncoding RNAs, collectively shaping the transcriptional landscape and phenotypic diversity of immune cells in response to viral antigens and inflammatory stimuli. DNA methylation involves the addition of methyl groups to cytosine residues within CpG dinucleotides, typically associated with transcriptional repression when occurring in gene promoter regions[14]. In the context of HIV infection, altered DNA methylation patterns at immune-related genes can influence T cell differentiation and effector functions[15]. Studies have shown that HIV infection in infants is associated with dysregulated DNA methylation profiles, affecting genes involved in immune activation pathways and cytokine production^[16,17]^. These epigenetic changes may contribute to sustained immune activation and impaired immune cell function observed in pediatric HIV/AIDS. Histone modifications, including acetylation, methylation, phosphorylation, and ubiquitination, regulate chromatin structure and accessibility, thereby controlling gene expression. During HIV infection, histone modifications at viral integration sites and host immune genes play critical roles in modulating immune responses[4]. Histone acetylation, for instance, is associated with active transcription of immune response genes and is crucial for the induction of antiviral immune responses. Conversely, histone methylation patterns can either activate or repress gene expression, depending on the specific lysine residues involved and the enzymatic context.

Noncoding RNAs, such as microRNAs (miRNAs) and lncRNAs, regulate gene expression post-transcriptionally and epigenetically. miRNAs can target mRNAs encoding key immune regulators and cytokines, influencing immune cell differentiation and function during HIV infection. Dysregulated expression of miRNAs has been implicated in promoting immune evasion strategies employed by HIV, such as downregulation of host antiviral defenses and immune activation pathways[18]. lncRNAs, on the other hand, can modulate chromatin structure and gene expression through interactions with chromatin-modifying complexes, thereby impacting immune cell phenotypes and responses to viral infections. The integration of these epigenetic mechanisms contributes to immune dysfunction observed in HIV-infected infants, characterized by chronic immune activation, impaired T cell responses, and compromised antiviral immunity. Epigenetic dysregulation may contribute to the establishment of viral reservoirs and persistence of HIV infection by altering host immune surveillance mechanisms and promoting viral latency[18].

### Impact of epigenetic dysregulation in HIV-infected infants

Epigenetic dysregulation in HIV-infected infants significantly alters the functionality of their developing immune system. One of the most notable impacts is the impaired maturation and activation of T cells. Histone modifications, such as increased histone deacetylation at loci encoding T-cell receptor signaling molecules, suppress the transcription of genes essential for T-cell activation and proliferation. This contributes to delayed immune responses and an increased vulnerability to opportunistic infections. Furthermore, altered histone methylation at immune checkpoint genes exacerbates T-cell exhaustion, a phenomenon marked by the inability of T cells to mount effective responses against HIV and other pathogens. Another critical consequence of epigenetic dysregulation in HIV-infected infants is the disruption of innate immune responses. For example, studies have shown that HIV induces hypomethylation of genes encoding pro-inflammatory cytokines in macrophages and monocytes, leading to chronic inflammation. This persistent inflammatory state damages tissues and further weakens the immune system. At the same time, hypermethylation of antiviral genes, such as those encoding interferons and pattern recognition receptors, inhibits the innate immune system’s ability to detect and respond to viral infections effectively[19].

B-cell function is also affected by epigenetic changes in HIV-infected infants. Epigenetic silencing of genes involved in immunoglobulin production reduces the capacity of B cells to generate effective antibodies, compromising the humoral immune response. For instance, hypermethylation of AICDA, a gene critical for class-switch recombination and somatic hypermutation, has been observed in HIV-infected infants, leading to suboptimal antibody diversity and efficacy. Epigenetic dysregulation also has a profound impact on immune memory formation. Noncoding RNAs, particularly microRNAs (e.g. miR-150 and miR-223), are dysregulated in HIV-infected infants and interfere with the differentiation of memory T and B cells. These changes hinder the establishment of robust adaptive immunity, leaving infants vulnerable to repeated infections. Additionally, disruptions in the epigenetic programming of innate immune cells, such as macrophages, impair their ability to develop trained immunity, a mechanism that enhances innate responses upon subsequent exposures to pathogens[20]. The chronic immune activation observed in HIV-infected infants is another consequence of epigenetic dysregulation. Hyperacetylation of promoters of inflammatory genes, such as TNF-α and IL-6, drives the overproduction of pro-inflammatory cytokines. This not only contributes to systemic inflammation but also accelerates immune cell turnover, depleting critical immune cell populations. In contrast, hypoacetylation of genes involved in immune regulation prevents the resolution of inflammation, perpetuating the cycle of immune dysfunction. HIV infection also affects the epigenetic landscape of the thymus, the site of T-cell development, in infants. Alterations in histone modifications and DNA methylation in thymic stromal cells disrupt the production of naive T cells, leading to a limited repertoire of T-cell receptors. This compromises the ability of the immune system to recognize and respond to diverse antigens, further impairing adaptive immunity[19]. Lastly, epigenetic changes in HIV-infected infants have been linked to long-term health consequences. Aberrant DNA methylation and histone modifications in genes associated with metabolic pathways contribute to the development of metabolic syndromes and growth impairments often observed in HIV-infected children. These findings underscore the broader systemic impact of epigenetic dysregulation beyond immediate immune dysfunction[20].

### Evidence linking methylation and epigenetic changes to HIV-associated accelerated aging

Emerging evidence highlights a strong association between epigenetic changes, particularly DNA methylation, and HIV-associated accelerated aging. Accelerated aging in HIV-infected individuals, including infants, is characterized by premature immune senescence, chronic inflammation, and increased susceptibility to age-related diseases. These phenomena are closely tied to alterations in epigenetic regulatory mechanisms.

1. DNA methylation and epigenetic clocks

HIV infection is associated with disruptions in DNA methylation patterns, which can be quantified using epigenetic clocks. These clocks estimate biological age based on methylation levels at specific CpG sites across the genome. Studies have consistently shown that HIV-infected individuals, including children, exhibit an increased biological age compared to their chronological age. This discrepancy indicates premature aging driven by epigenetic dysregulation. For instance, methylation at CpG sites associated with the DNAm PhenoAge and DNAm GrimAge clocks has been shown to accelerate in HIV-positive populations, linking these changes to inflammation, immune senescence, and mortality risk[21].

2. Hypomethylation of pro-inflammatory genes

HIV-associated accelerated aging is partly driven by chronic immune activation and systemic inflammation, which are underpinned by epigenetic changes. Hypomethylation of pro-inflammatory gene promoters, such as TNF-α, IL-6, and IFN-γ, has been observed in HIV-infected individuals. This hypomethylation leads to sustained overexpression of these cytokines, perpetuating a state of “inflammaging” – chronic, low-grade inflammation characteristic of aging[22].

3. Hypermethylation of DNA repair and telomere maintenance genes

DNA repair and telomere maintenance are critical processes that decline with age and are exacerbated in HIV infection due to epigenetic dysregulation. Hypermethylation of genes involved in DNA repair pathways, such as BRCA1 and ATM, impairs the cellular ability to repair DNA damage, resulting in genomic instability. Similarly, hypermethylation of telomere-related genes, including TERT, which encodes the catalytic subunit of telomerase, leads to shortened telomeres. Telomere attrition is a hallmark of aging and is more pronounced in HIV-infected individuals[23].

4. Epigenetic silencing of mitochondrial function genes

HIV infection is linked to mitochondrial dysfunction, a key driver of cellular aging. Epigenetic silencing of nuclear-encoded mitochondrial genes, such as those regulating oxidative phosphorylation, has been documented in HIV-infected individuals. This silencing, mediated by hypermethylation of gene promoters, results in impaired energy production, increased oxidative stress, and cellular senescence[24].

5. Immune cell-specific methylation patterns

In HIV-infected infants, immune cells exhibit distinct methylation patterns that mirror those seen in older individuals. For example, naive T cells, which are critical for mounting effective immune responses, show increased methylation of genes associated with T-cell activation and differentiation. This epigenetic repression accelerates the transition of the immune system toward a memory phenotype, a process typically associated with aging.

6. Accelerated epigenetic aging in pediatric populations

Although most studies on HIV and epigenetic aging have focused on adults, emerging evidence in pediatric populations reveals similar trends. HIV-infected infants and children on antiretroviral therapy (ART) exhibit DNA methylation changes that align with accelerated epigenetic aging. These alterations are particularly evident in genes involved in inflammation, immune regulation, and cellular metabolism. For instance, methylation at loci within the HLA region, which plays a role in immune function, is significantly altered in HIV-positive children, correlating with markers of aging[25].

7. Reversibility of epigenetic changes

Some studies suggest that epigenetic changes associated with accelerated aging may be partially reversible with effective ART. However, ART alone does not fully normalize methylation patterns or eliminate the risk of premature aging (Table 1). This indicates that additional interventions, such as epigenetic therapies targeting specific methylation changes, may be necessary to mitigate aging-related effects in HIV-infected populations^[26,27]^.

**Table 1.** Summary of key epigenetic modifications observed in HIV-infected infants.

Epigenetic modification	Description	Affected immune cells	Impact on immune function
DNA methylation	Hypermethylation of promoter regions in immune-related genes	T cells, monocytes	Reduced gene expression of antiviral responses
Histone acetylation	Decreased acetylation at H3K27 in pro-inflammatory genes	CD4+ T cells	Impaired cytokine production and T-cell activation
Noncoding RNA (miRNA)	Upregulation of miR-146a in response to chronic inflammation	Monocytes, Macrophages	Inhibition of innate immune signaling pathways
Chromatin remodeling	Altered chromatin accessibility at HIV integration sites	CD4+ T cells, Dendritic Cells	Maintenance of viral latency and immune evasion

CD4, cluster of differentiation 4.

### Clinical implications and therapeutic strategies

The clinical implications of epigenetic dysregulation in HIV-infected infants extend beyond immune dysfunction to encompass disease monitoring, treatment responses, and long-term health outcomes. Epigenetic biomarkers have emerged as valuable tools for monitoring disease progression and predicting treatment outcomes in HIV-infected infants[28]. DNA methylation patterns at specific gene loci, histone modification profiles, and noncoding RNA expression levels can serve as indicators of immune activation, viral reservoir dynamics, and host response to ART. Integrating epigenetic biomarkers into clinical practice may facilitate early detection of treatment failure, guide therapeutic decisions, and optimize disease management strategies to enhance patient outcomes. Epigenetic insights provide a basis for developing personalized treatment approaches in pediatric HIV/AIDS[29]. Targeting epigenetic regulators, such as histone deacetylases and DNA methyltransferases, with pharmacological inhibitors or gene-editing technologies holds promise for modulating immune responses, reducing viral reservoirs, and enhancing immune reconstitution in affected infants. Combining epigenetic therapies with conventional ART regimens may improve treatment efficacy, minimize drug resistance, and mitigate long-term complications associated with HIV infection[21]. Epigenetic therapies aimed at reversing HIV-induced epigenetic alterations offer potential benefits for enhancing immune reconstitution in HIV-infected infants[30]. Restoring normal DNA methylation patterns and histone modification profiles at immune gene loci can promote T cell differentiation, cytokine production, and immune surveillance capabilities essential for controlling viral replication and preventing opportunistic infections.

#### Current therapeutics and proposed improvements

##### Current therapeutic approaches

###### Antiretroviral therapy

ART remains the cornerstone of HIV management in infants, effectively suppressing viral replication and reducing mortality. While ART has drastically improved survival rates, it does not fully restore immune function, nor does it eliminate the virus, which persists in latent reservoirs. Additionally, ART has limited impact on reversing the epigenetic modifications induced by HIV infection^[31^-^34]^.

### Epigenetic modifiers

Emerging research suggests that epigenetic modifiers, such as DNMTis (e.g. azacitidine) and HDACis (e.g. vorinostat), could potentially reverse HIV-induced epigenetic changes. These agents work by altering the epigenetic landscape, potentially reactivating silenced antiviral genes and reducing the size of viral reservoirs. However, their application in infants is still in its infancy, with significant concerns regarding safety, specificity, and long-term effects[25].

### Immunomodulatory therapies

Immunomodulatory therapies that target epigenetic pathways are also being explored. These include cytokine-based treatments that aim to correct the dysregulated immune environment caused by HIV. For example, IL-7 therapy is being studied for its potential to enhance T-cell function and reduce T-cell exhaustion, partly mediated by epigenetic mechanisms.

## Limitations and challenges

### Safety and efficacy of epigenetic therapies

One of the primary challenges in using epigenetic therapies in HIV-infected infants is ensuring safety and efficacy. Infants have developing immune systems and organs, making them particularly vulnerable to the potential off-target effects of epigenetic drugs. These therapies could unintentionally alter the expression of critical genes involved in growth and development, leading to unforeseen consequences. Long-term studies are needed to assess the safety of these therapies in pediatric populations[21].

### Specificity of epigenetic modifiers

Epigenetic modifiers, while promising, often lack specificity. Drugs like DNMTis and HDACis can affect a wide range of genes, not just those involved in HIV pathology. This lack of specificity raises concerns about unintended epigenetic changes that could disrupt normal cellular functions and contribute to toxicity. Developing more targeted epigenetic therapies that specifically modulate HIV-related pathways is a significant challenge.

### Resistance and viral reservoirs

Another challenge is the potential for resistance to epigenetic therapies, similar to what is observed with ART. The ability of HIV to persist in latent reservoirs despite aggressive treatment remains a significant barrier to curing the infection. Epigenetic therapies aimed at reactivating these reservoirs might only have limited success, as the reactivated virus could still evade immune detection or develop resistance to the therapeutic agents. This highlights the need for combination strategies that include both ART and epigenetic modulators.

### Biomarker development

A major limitation in the clinical application of epigenetic therapies is the lack of reliable biomarkers to monitor therapeutic efficacy. There is a need to develop biomarkers that can accurately reflect changes in the epigenetic landscape and correlate with immune function improvement. Without such markers, it is difficult to optimize treatment regimens or predict patient outcomes.

### Ethical and regulatory considerations

The use of epigenetic therapies in infants raises ethical and regulatory challenges. Given the experimental nature of these treatments and the potential for long-term consequences, careful consideration must be given to the risks and benefits. Regulatory agencies will require robust evidence from preclinical and clinical studies before approving these therapies for pediatric use.

### Future directions

Future directions in the study of epigenetic modifications in HIV-infected infants hold promise for advancing our understanding of disease pathogenesis, improving therapeutic strategies, and ultimately achieving better outcomes for affected children. Here are some key areas for future research and development:

1. Precision medicine approaches: Tailoring treatment strategies based on individual epigenetic profiles holds great potential for optimizing therapeutic outcomes in pediatric HIV/AIDS[23]. Future research should focus on identifying epigenetic biomarkers that predict disease progression, treatment responses, and susceptibility to complications. Integrating multi-omics data (epigenomics, transcriptomics, proteomics) and employing artificial intelligence (AI) algorithms may enhance our ability to personalize treatment regimens and implement precision medicine approaches that consider individual variations in epigenetic regulation.
2. Epigenetic editing technologies: Advancements in genome editing technologies, such as CRISPR-Cas9, offer new opportunities for targeted manipulation of epigenetic marks associated with HIV infection[24]. Future studies should explore the feasibility and safety of using epigenetic editing tools to modulate immune responses, enhance viral clearance, and potentially achieve functional cure in pediatric populations. Developing robust delivery systems and ensuring specificity and efficacy of epigenetic editing tools will be critical for translating these technologies into clinical applications^[27,28]^.
3. Longitudinal studies and biomarker validation: Longitudinal studies are essential for tracking changes in epigenetic profiles over time and assessing their correlation with clinical outcomes in HIV-infected infants. Future research should prioritize longitudinal cohort studies that integrate comprehensive epigenetic analyses with clinical data to validate the predictive value of epigenetic biomarkers for disease progression, treatment responses, and long-term health outcomes. Establishing standardized protocols for sample collection, analysis, and data interpretation will facilitate comparison across studies and enhance the reliability of epigenetic biomarkers in clinical practice.
4. Epigenetic regulation of immune memory: Investigating the role of epigenetic modifications in shaping immune memory responses to HIV and other infections is crucial for developing effective vaccination strategies and enhancing immune protection in pediatric populations. Future research should explore how epigenetic marks acquired during early life influence immune memory formation, durability, and responsiveness to vaccination[21].
5. Addressing epigenetic plasticity and environmental influences: Epigenetic modifications are dynamic and responsive to environmental factors, including prenatal exposures, nutrition, and socioeconomic conditions. Future studies should investigate how environmental influences during critical developmental windows impact epigenetic programming and immune responses in HIV-infected infants. Integrating environmental epigenomics with clinical epidemiology may elucidate gene-environment interactions that modulate susceptibility to HIV infection, disease progression, and treatment outcomes.
6. Translational research and clinical trials: Translating findings from basic epigenetic research into clinical applications requires rigorous evaluation through well-designed clinical trials and collaborative efforts between researchers, clinicians, and industry partners. Future directions should prioritize conducting randomized controlled trials to assess the safety, efficacy, and long-term outcomes of epigenetic-based therapies in pediatric HIV/AIDS. Establishing regulatory frameworks and ethical guidelines for conducting epigenetic clinical trials will be essential for ensuring patient safety and regulatory approval of novel therapeutic interventions.

## Conclusion

Epigenetic modifications play a crucial role in shaping the immune responses of HIV-infected infants, significantly impacting disease progression and therapeutic outcomes. Alterations in DNA methylation, histone modifications, and noncoding RNA expression can lead to impaired immune function, influencing the infant’s ability to control HIV infection. The potential of epigenetic therapies, such as DNA methylation inhibitors, HDACis, and miRNA modulators, offers promising avenues for improving immune responses and controlling HIV in pediatric populations. These therapies aim to correct dysregulated gene expression, enhance immune function, and reduce viral reservoirs, thereby improving the overall health and prognosis of HIV-infected infants.

## Data Availability

Not applicable as this is a review.

## Research registration unique identifying number (UIN)

Not applicable as this is a review.
